# Electroencephalography complexity in resting and task states in adults with attention-deficit/hyperactivity disorder

**DOI:** 10.1093/braincomms/fcac054

**Published:** 2022-03-07

**Authors:** Chao Gu, Zhong-Xu Liu, Steven Woltering

**Affiliations:** 1 Department of Neuroscience, Texas A&M University, College Station, TX, USA; 2 Department of Psychiatry, Massachusetts General Hospital, Boston, MA, USA; 3 Department of Behavioral Sciences, University of Michigan-Dearborn, Dearborn, MI, USA; 4 Department of Educational Psychology, Texas A&M University, College Station, TX, USA; 5 Department of Applied Psychology and Human Development, University of Toronto, Toronto, Canada

**Keywords:** EEG, multiscale entropy, ADHD, resting state, task state

## Abstract

Analysing EEG complexity could provide insight into neural connectivity underlying attention-deficit/hyperactivity disorder symptoms. EEG complexity was calculated through multiscale entropy and compared between adults with attention-deficit/hyperactivity disorder and their peers during resting and go/nogo task states. Multiscale entropy change from the resting state to the task state was also examined as an index of the brain’s ability to change from a resting to an active state. Thirty unmedicated adults with attention-deficit/hyperactivity disorder were compared with 30 match-paired healthy peers on the multiscale entropy in the resting and task states as well as their multiscale entropy change. Results showed differences in multiscale entropy between individuals with attention-deficit/hyperactivity disorder and their peers during the resting state as well as the task state. The multiscale entropy measured from the comparison group was larger than that from the attention-deficit/hyperactivity disorder group in the resting state, whereas the reverse pattern was found during the task state. Our most robust finding showed that the multiscale entropy change from individuals with attention-deficit/hyperactivity disorder was smaller than that from their peers, specifically at frontal sites. Interestingly, individuals without attention-deficit/hyperactivity disorder performed better with decreasing multiscale entropy changes, demonstrating higher accuracy, faster reaction time and less variability in their reaction times. These data suggest that multiscale entropy could not only provide insight into neural connectivity differences between adults with attention-deficit/hyperactivity disorder and their peers but also into their behavioural performance.

## Teaser

Gu *et al*. found that the brains of individuals with attention-deficit/hyperactivity disorder are less variable when comparing passive to active mental states than the brains of their peers. Findings increase our understanding of different mental states and flexibly shift between them in attention-deficit/hyperactivity disorder.

## Graphical Abstract

**Graphical Abstract:**
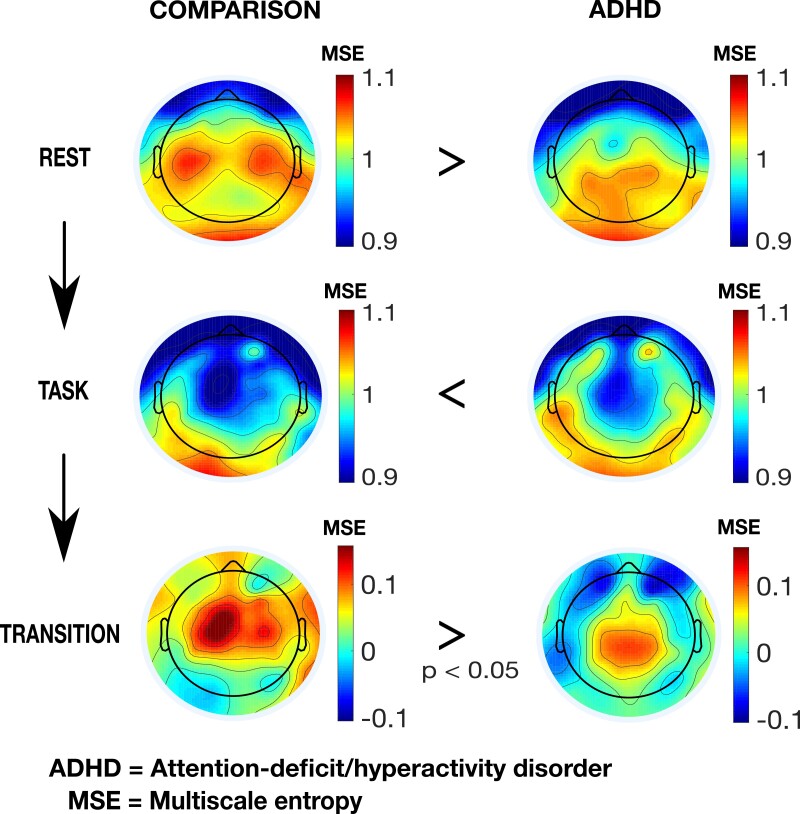

## Introduction

Attention-deficit/hyperactivity disorder (ADHD) is among the most common mental disorders in children and has a negative influence on children’s daily life and school performance.^1–3^ Furthermore, symptoms and negative effects of ADHD on an individual’s life often remain when they grow up.^4,5^ The arrival of adulthood would also bring new areas of cognitive impairment.^6^

Studies show that individuals with ADHD have difficulty with cognitive flexibility, which generally refers to individuals’ ability to adapt flexibly to changing environments or switch smoothly between mental states.^7–10^ Abnormalities of cognitive flexibility could even help explain deficits in inhibition control and working memory, which were usually associated with ADHD.^11,12^

A recently used electrophysiological (EEG) measure that may be sensitive to an individual’s mental states and capacity to flexibly switch between them, is EEG complexity.^13^ EEG complexity, which is a variability measurement of the dynamic spatial or temporal patterns of the EEG signal, has proven valuable in characterizing brain activity between individuals during cognitive tasks and at rest^14–16^ and it has been connected to cognitive processes, such as sensory differentiation, learning and decision making.^17^ As such, EEG complexity may be a measure that is sensitive in discriminating between individuals with and without ADHD and may prove helpful in better understanding the deficits that individuals with ADHD struggle with, like cognitive flexibility.

A number of theories explain the functionality of EEG complexity, such as dynamic range,^18^ Bayesian optimization^19^ and Itinerant dynamics theory.^20^ In brief, dynamic range theory^21^ states that increased brain signal variability would indicate a greater dynamic range of behaviour, which implies the brain could respond more effectively to a larger range of stimuli. Under the theory of Bayesian optimality,^19^ increased brain signal variability relates to a broader probability distribution for networks to make optimal responses. Finally, in itinerant dynamic theory,^20^ variability dynamics of brain activity reflect a tendency not to settle to any particular state but to explore from one state to the next across moments, which is described as itinerancy. Increased variability in brain activity suggests larger itinerancy of the neural system which could translate into more flexible formation and utilization of functional modules. We refer to the review by Garrett *et al*.^22^ and Waschke *et al*.^23^ for a more detailed overview of these theories. In the current paper, we will mainly adopt the itinerancy theory as it relates to attention.

Brain complexity can be measured during different mental states. Traditionally, brain activity has been divided into tonic and phasic activity.^24^ The tonic activity represents the brain’s default activity and provides the substrate for brain function,^25^ which is typically studied through spontaneous brain activity during resting states.^26^ The spontaneous brain activity measured on the scalp during resting states, noted as tonic activity, consists of electrical activity from distributed sub-networks.^27^ Each sub-network is representing a functional module and the configuration of these functional modules can be seen as a representation of the spontaneous brain network.^28^ Spontaneous brain activity has unique spatial–temporal patterns that instruct neural functioning.^29^

Phasic activity represents the stimulus-driven brain activity which is a relatively small portion of brain activity compared with the overall brain activity.^22,30^ This is typically studied through brain activity during tasks.

During active task states, the evoked brain activity measured on the scalp contains tonic activity and phasic activity, and the phasic activity is the stimulus-driven activity operating on existing tonic activity.^22^ The evoked brain activity is constructed by electrical activity from distributed functional sub-networks, and the overall activation has unique patterns that may instruct cognitive functioning and task requirements.^31–33^

EEG complexity in resting and task states may represent the itinerancy of the spontaneous and evoked brain activity, respectively. The itinerancy under such different mental states could relate to the degree of cognitive flexibility since cognitive flexibility can be understood as reflective of a set of neural properties that facilitate flexible switching between different mental states. The itinerancy of the brain, as a non-linear system spontaneously exploring the state space of possible thought and action tendencies, may then reflect its level of wandering across states. This can be true during task activity, when the itinerancy is perhaps more constrained due to stimuli, versus the more mind-wandering internal states experienced during rest.

The present study constitutes the first comprehensive exploration of the difference in multiscale entropy (MSE), as an indicator of EEG complexity, between young adults with ADHD and their healthy peers. We intended to calculate MSE and evaluate the itinerancy of the brain across the resting and active task states and estimate their relationship with ADHD patients’ cognitive deficits.

MSE measures the temporal complexity by estimating the sample entropy of the data downsampled by different time scales.^34,35^ Sample entropy captures the unpredictability of the data and evaluates the appearance of repetitive patterns.^36,37^ MSE accesses the unpredictability in the data over multiple time scales, which assigns high values to random signals and low values to highly deterministic signals. MSE can evaluate both high-frequency temporal complexity over fine scales (1–6) and low-frequency temporal complexity over coarse scales (15–20), which could reveal local information processing and long-range interaction between different sites respectively.^38–40^

MSE during resting and active task states has been related to several important features of the human condition, such as aspects of neural development, ageing, cognitive functioning and psychopathology.^15,41–44^ MSE in the resting and active task states increased with the maturation of the brain at an early age,^16,45,46^ which decreased with the shrinkage and cortical thinning of the brain in ageing.^47,48^ There’s concrete evidence suggesting that individuals with Mild Cognitive Impairment had lower MSE than their healthy peers and higher MSE than patients with Alzheimer’s disease.^43^ Bilingual individuals were found to have higher MSE than monolinguals in occipital sites during a task-switching experiment.^37^ Related to mental health, EEG complexity from individuals with Autism disorder was lower than that from healthy controls during resting and active task states.^41,44^

To the best of our knowledge, few studies have applied MSE to examine ADHD. In a study by Chenxi *et al*.,^49^ 13 children diagnosed with ADHD and their healthy peers were recruited to conduct multi-source interference tasks. MSE in the delta and theta frequency bands was higher among children with ADHD compared with their peers, while MSE in the alpha frequency band was lower. These findings suggested MSE across different frequency bands could reveal abnormal EEG patterns associated with ADHD. In Boroujeni *et al*.,^50^ sample entropy was successfully used as one of the neural features to train classifiers for ADHD diagnosis on 50 children with ADHD and 26 healthy participants.

Although group differences in MSE patterns between children with ADHD and control groups have been found,^49^ differences in MSE between adults with ADHD and their peers have not been explored. Moreover, to the best of our knowledge, within-subjects changes of MSE between the resting and active task states, which could reveal the transition between tonic activity and phasic activity,^24,51,52^ have not been investigated.

In the current study, college students participated in resting-state sessions and a go/nogo task,^53,54^ while EEG was recorded to investigate the brain signal complexity. By calculating and analysing MSE across different cognitive states, our study not only aims to determine if MSE could be a promising candidate as a neurocognitive indicator of adult ADHD but can also interpret executive function deficits in ADHD with MSE in different mental states.

Based on our previous finding that participants with ADHD showed lower inter-trial variability in EEG oscillation power,^53^ we hypothesized that in the resting state, the MSE from the ADHD group would be lower than that from the control group, which may indicate smaller itinerancy in the tonic activity from participants with ADHD and may be related to their difficulty in initializing and disengaging attention in a flexible manner. In the task state, we anticipated that the MSE from the ADHD group would be lower than that from the ADHD group, which may suggest lower itinerancy in the evoked activity from ADHD participants. This could explain their difficulty in switching between different trials of tasks. Finally, from the resting state to the task state, we expected larger differences in MSE transitions in the control group, which suggested that the brains of healthy individuals have a better ability to effectively switch between different mental states.

## Materials and methods

### Participants

Data were taken from a larger study investigating changes in neural and behavioural indices after a working memory training programme conducted at the University of Toronto from 2011 to 2013 (see also Mawjee *et al*.^55^; Woltering *et al*.^56^). Data for this study were taken from pre-training visits only. For this study, 30 unmedicated college students with ADHD were pair-matched with 30 peers on gender (15 males, 15 females) and age [mean age 23, standard deviation (SD) = 3]. We refer to the supplements for a more detailed breakdown of how the present sample was selected.

Students with ADHD were recruited from the University Student Service, while 30 healthy control students (15 males, 15 females; mean age = 23, SD = 3.4) were recruited through campus advertisements. Inclusion criteria for the ADHD group were (i) a previous diagnosis of ADHD and (ii) registration at the College Student Disability Services, which required supporting documentation proof.

All participants were asked to finish the Adult ADHD Self Report Scale (ASRS v1.1^57^) to assess their current symptoms of ADHD. Exclusion criteria (for control and ADHD group) were defined as (i) uncorrected sensory impairment; (ii) major neurological dysfunction; (iii) mood affecting medication rather than a prescription for ADHD. We also used the Cognitive Failures Questionnaire (CFQ) to support our classification of ADHD subjects. Participants in the ADHD group had evidence of a Diagnostic and Statistical Manual of Mental Disorders (DSM)-IV diagnosis of ADHD as provided by the university student disability services.

### Procedure

Participants came to the laboratory to complete a behavioural assessment and several computerized tasks with EEG. The behavioural assessment was conducted first and included a battery of neuropsychological tests and behavioural rating scales which lasted up to 3 h (see Mawjee *et al*.^55,58^ for details). After a break, the EEG assessments began. Participants were asked to sit in front of a screen and complete tasks while wearing a 129-electrode EEG net (Electrical Geodesic Inc., EGI). After becoming familiar with the environment, instructions on a screen explained the task to participants. EEG tasks included, in order, a resting-state task,^53^ a change detection task,^12^ a delayed match-to-sample working memory task,^59,60^ a go/nogo task,^54,61^ and finally, another resting-state task. Participants typically took a 10–15 min break before the EEG tasks started and also took short breaks in between tasks. We also note that, with the exception of the go/nogo task, the other EEG tasks were self-paced and allowed participants to take short breaks even during the tasks.

The study was approved by the institutional research ethics board at the University of Toronto (Protocol reference: #23977) and the original clinical trial was pre-registered (#NCT01657721). Participants received $20 for their participation in the pre-visit (and $150 if they completed the post-training visit). Informed written consent was obtained from all participants prior to beginning the assessments.

### EEG measures

#### Resting-state EEG

The resting state consisted of six 40-s intervals (i.e. 240 s in total and 120 s for eyes-opened or eyes-closed conditions). Before each interval, a sound signalled when they were to alternate closing or opening their eyes. To avoid potential artefacts from eye movement and based on prior research showing the eyes-closed condition to be most sensitive to group differences,^53^ we focused on intervals under eyes-closed conditions. Furthermore, data from the first resting state were used for this study to avoid conflation of potential fatigue effects in the second resting state after the tasks.

#### Go/nogo EEG task

The go/nogo task, which is identical to the one reported in Woltering *et al*.^54^ and Liu *et al*.,^61^ was presented by E-prime software (Psychological Software Tools, Pittsburgh, PA, USA). Participants were instructed to press a button as soon as a letter appeared on the screen during the go condition and hold their response when a letter was repeated for the second time during the nogo condition. Participants finished a practice block of 21 trials before the actual task started. In our study, we decided to focus on the correct go trials, which reflected a more action-oriented state and accounted for the majority of go/nogo trials.

Several behaviour measurements of the go/nogo task were computed to evaluate participants’ performance, including reaction time (RT), accuracy (ACC) and standard deviation of reaction time (RTSD), each of which was extracted by E-prime software.

### Clinical measures

All participants were asked to complete a series of standard questionnaires and tasks to evaluate their current levels of behavioural, cognitive and social emotional functioning.

#### Adult ADHD Self Report Scale

The ASRS is a valid and reliable measurement for evaluating ADHD symptoms in adults, which has 18 questions based on the criteria used for ADHD diagnosis in the DSM-V.^62^ The ASRS Part A has been found to be most predictive of symptoms consistent with a diagnosis of ADHD. Scores for these questions were added up to calculate a final score in the current paper. For original inclusion purposes, we used a modified version of the six-item ASRS screener which was administered by telephone (see Gray *et al*.,^63^ for more information on this modified version and its psychometric properties).

#### Cognitive Failures Questionnaire

The CFQ measures self-reported failures in perception, memory and motor function in everyday life. There are 25 questions, which ask participants to rank how often these mistakes occur.^64^ Its reliability and validity in quantifying the distractibility of individuals have been established in previous research.^65,66^

### EEG data acquisition, data processing and MSE calculation

#### Data acquisition

Participants were seated in a comfortable chair. The EEG data were acquired using a 128-channel sensor net (Electrical Geodesic, Inc., Eugene, OR, USA). Impedances for all channels were set below 50 kΩ which is commonplace for high-impedance systems and recommended for child and clinical populations due to fast application (see Brooker *et al*.^33^ and Ferree *et al*.^67^). The data were collected using a 0.1–1000 Hz bandpass hardware filter, a 500 Hz sampling rate, EGI Netstation stand-alone software and referenced to electrode Cz.

#### Data processing

EGI’s Netstation software was used to filter (0.5–50 Hz) and segment the EEG data. For resting states, EEG data were segmented into 2-s epochs. For task states, EEG data were segmented into 1.4-s epochs (400 ms before stimulus onset, 1000 ms post-stimulus). Average referencing was applied after the data segmentation. Then data were transferred into MATLAB for further analysis.

To remove artefacts from the EEG data, such as eyeblink or muscle movement, we utilized the Independent Component Analysis (ICA) procedure in EEGLAB. The EEG data were decomposed into independent components and automatically identified for brain activity, eye movement, muscle artefact and other artefacts by the SASICA package.^68^ Then one research assistant reviewed the results from SASICA and made adjustments for the definition of EEG components to remove EEG contributions from artefactual sources. On average, there were about 15% of ICA components removed for the resting-state task, while about 28% of ICA components were removed for the go/nogo task state.

For the resting-state task, there were about 7% trials rejected, while about 13% trials were rejected for the go/nogo task. On average, there are 56 resting artefact-free trials (SD = 4) and 149 correct go artefact-free trials (SD = 4) in the comparison group, while there are 55 resting (SD = 3) and 155 correct go artefact-free trials (SD = 24) in the ADHD group. There was no difference in trial count between the groups as tested using a Kruskal–Wallis test for the resting as well as go/nogo task state. We applied the same preprocessing protocol for data recorded in the resting and task states.

#### MSE calculation

After the EEG was cleaned, MSE was calculated for each trial at each electrode over corresponding scales. The calculation of MSE was done by an open-source algorithm available at www.physionet.org/physiotools/mse/. Parameters for *m* and *r* were chosen on the basis of the size of our segmentation, which were 700 time points^16^ and Costa *et al*.^69^ Parameter values *m* = 2, *r* = 0.5, were used to calculate the sample entropy of the data at different time scales.^28,70^ Scale refers to the length of the window used in the coarse-graining procedure and can provide an indication of temporal resolution of processing with distributed processes often reflected by activity in larger timescales versus local processing. We calculated MSE over Scales 1–20, which we grouped into three ranges: a fine scale (Scale 1–6), mid scale (Scale 7–14) and coarse scale (Scale 15–20). The calculation procedure includes two steps: (i) obtain coarse-grained EEG data by averaging the data points within non-overlapping windows of length *t*, which is corresponding to time scale; (ii) calculate sample entropy for each coarse-grained data series, which measures the regularity by evaluating the appearance of repetitive patterns.

MSE in the resting state was calculated over fine scale, mid scale and coarse scale,^71^ while MSE in the task state was calculated for the fine and mid scales due to the shorter duration of the segment. To observe how the brain adapts to the task, the MSE transition (MSE-Δ) was obtained by subtracting MSE in the task state from MSE in the resting state over corresponding scales (Fine and Mid) and electrodes. In terms of the montage, the 10–10 system was utilized to group electrodes into five clusters at the left and right hemispheres correspondingly.^72^ Each cluster of electrodes represented one brain site as shown in Supplementary Fig. 1. MSE and MSE-Δ were averaged within each cluster and checked for outliers.

### Statistical analysis

We averaged MSE measurements (MSE in the resting and task states and MSE transition MSE-Δ) across electrodes within different brain sites and scale ranges. Data points located outside two SDs from the mean for averaged MSE measurements within each group were identified as outliers and removed. After removing outliers, we paired participants between the healthy control and ADHD groups. No violations of normality were found using the Shapiro–Wilk test as applied for each MSE measurement in each group. Levene’s Test was used to check the homogeneity of variances for each MSE measurement. No violations were reported. A three-way mixed measure ANOVA was used to examine the effects of Scale (Fine, Mid and Coarse) and Site (Frontal, Temporal, Central, Parietal, Occipital) as within-subject factors and Group (COMP and ADHD) as a between-subjects factor on these MSE measurements. When justified, comparisons between factors were further explored using independent *t*-tests. To examine the relationship between behavioural performance and MSE measurements, Pearson’s *r* correlation coefficients were utilized.

Partial eta-squared were computed to verify the effect size. According to Vacha-Haase and Thompson,^73^ partial *η*^2^ = 0.01 corresponds to a small effect, partial *η*^2^ = 0.10 standards for a medium effect and partial *η*^2^ = 0.25 represents a large effect.

### Data availability

The data that support the findings of this study are available from the corresponding author, upon reasonable request.

## Results

### Behavioural and questionnaire measures

The means and SDs of the questionnaire and behavioural measurements were compared between the ADHD group and their peers (see Table 1). Results showed that the ADHD group reported significantly more symptomatology and cognitive problems than their peers. The ADHD group also showed significant differences from their peers in having lower go-trial ACC, as well as higher intra-individual variability reflected by higher RTSD in the go trials during the task.

**Table 1:** Descriptive and group differences for questionnaire and behavioural measurements

	COMP		ADHD		Group difference	Effect size
	*M*	SD	*M*	SD	*P*	*η* ^2^
ASRS	21.87	9.15	48.53	10.03	<0.001**	0.666
CFQ	27.77	9.30	56.50	13.87	<0.001**	0.605
GO ACC	0.93	0.05	0.89	0.06	<0.05*	0.095
GO RT	304.43	31.11	315.20	31.67	0.190	0.029
GO RTSD	85.26	15.88	101.50	16.00	<0.001**	0.212

COMP is the comparison group; ADHD is the ADHD group; ASRS is the Adult ADHD Self-Report Scale; CFQ is the cognitive failures questionnaire; GO ACC is the accuracy in the correct go trials; GO RT is the reaction time in the correct go trials; GO RTSD is the standard deviation of reaction time in the correct go trials. *M* and SD are used to represent the mean and SD, respectively. The *P*-value of group difference is calculated by paired two-tailed *t*-test.

* *P* < 0.05.

** *P*  <  0.001.

### MSE in resting state

Our first hypotheses predicted that the MSE from the comparison group would be larger than that from the ADHD group.

**Figure 1:**
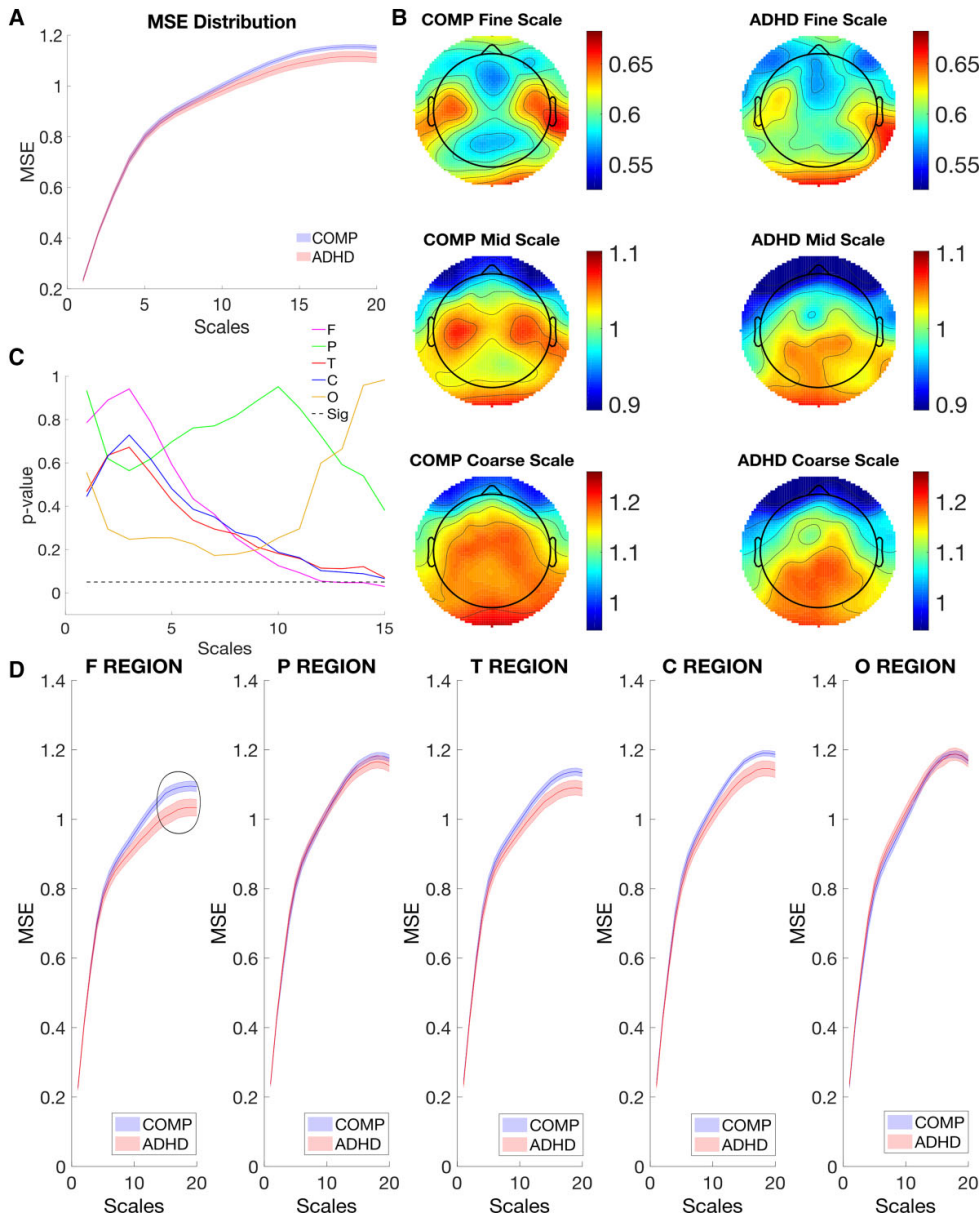
**Temporal and spatial comparison of MSE in the resting state of EEG.** (**A**) The comparison of MSE over scales. MSE from the COMP and ADHD groups were averaged across all sites and compared over scales, with standard error as a shaded area. (**B**) The topographic comparison of MSE. MSE was averaged over all scales and compared topographically. (**C**) Group difference of MSE between the COMP and ADHD groups. A paired *t*-test of MSE averaged within each site was performed over all scales. The Benjamini and Hochberg method (FDR = 0.05) was applied to adjust the *P*-values, which was not significant in the resting state. (**D**) Comparison of MSE over scales within different sites in the resting state. MSE was averaged within each site and compared over scales with standard error as a shaded area. *Note*: MSE is the multiscale entropy; F Site is the frontal site; P Site is the parietal site; T Site is the temporal site; C Site is the central site; O Site is the occipital site.

First, significant main effects were found for Scale [*F*(2, 116)  =  1979.945, *P* < 0.001, *η*^2^ = 0.972] and Site [*F*(4, 232)  =  42.46, *P* < 0.001, *η*^2^ = 0.423], which suggested MSE was different at different scales or sites as shown in Supplementary Table 1. To illustrate MSE difference, we plotted MSE across different scales, which indicated MSE increased with scale in both comparison and ADHD groups as shown in Fig. 1A. MSE in frontal sites was lower than that in other sites (Supplementary Table 1). The main effect of Group was not significant [*F*(1, 58) = 1.507, *P* = 0.225, *η*^2^ = 0.025].

However, the results of the repeated measure ANOVA also showed a significant three-way interaction among Group, Site and Scale [*F*(8, 464)  =  2.937, *P* < 0.01, *η*^2^ = 0.048], indicating that MSE between the two groups differed at different sites depending on Scale, as illustrated in Fig. 1B. To examine MSE in a more detailed manner, we compare MSE at different sites over different sites.

We also found a statistically significant two-way interaction between Site and Group [*F*(4, 232)  =  4.410, *P* < 0.01, *η*^2^ = 0.071]. The results indicated that when all scales were combined, differences of MSE between the comparison and ADHD groups were found at frontal sites, while similar MSE distributions at other sites (Fig. 1D). The two-way interaction between Scale and Site [*F*(8, 464)  =  96.422, *P* < 0.001, *η*^2^ = 0.624] was also found statistically significant, which suggested, MSE has different distribution over scales at different sites in both groups. The two-way interaction between Scale and Group [*F*(2, 116)  =  1, 725, *P* = 0.183, *η*^2^ = 0.029] was not statistically significant.

Despite the significance of the main Omnibus ANOVA, further Benjamini and Hochberg^74^ corrections, i.e. the false discovery rate (FDR) = 0.05, to adjust *P*-values indicated that none of the *post hoc* effects survived significance. We will therefore interpret these findings with caution. Our findings confirmed our hypothesis, albeit weakly, indicating that MSE for the comparison group was found to be larger than that of the ADHD group at frontal sites over coarse scales (Fig. 1A, C and D).

**Figure 2:**
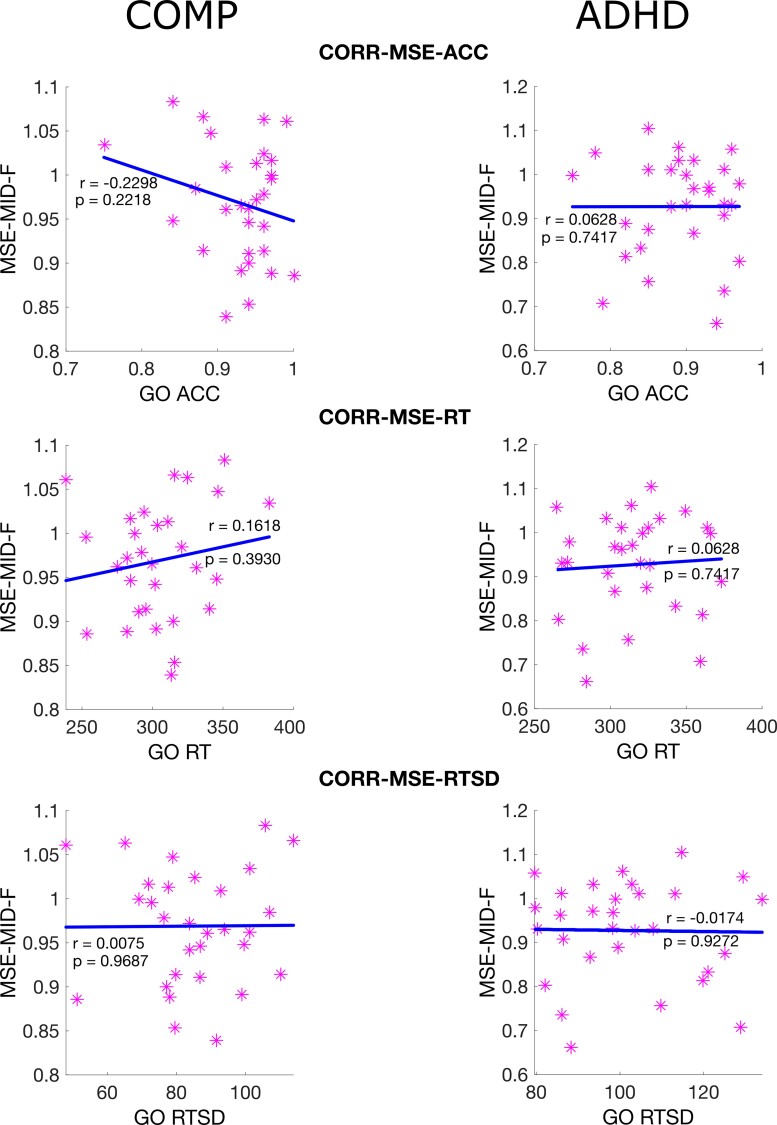
**Correlation between MSE in the resting state and task performance.** Scatter plots with a regression line between MSE averaged over mid scales at the frontal site and three task performance measurements, including ACC for go task, RT for go task and RTSD for go task. *Note*: CORR-MSE-ACC is the correlation between MSE and the ACC; CORR-MSE-ACC is the correlation between MSE and the RT; CORR-MSE-RTSD is the correlation between MSE and the RTSD; COMP is the comparison group; ADHD is the ADHD group.

Finally, no statistically significant relationship was found between MSE and behaviour measurements of the go/nogo task in the comparison or ADHD groups as shown in Fig. 2.

### MSE in the task state

Our second hypothesis stated that the MSE from the COMP group would be larger than that from the ADHD group.

**Figure 3:**
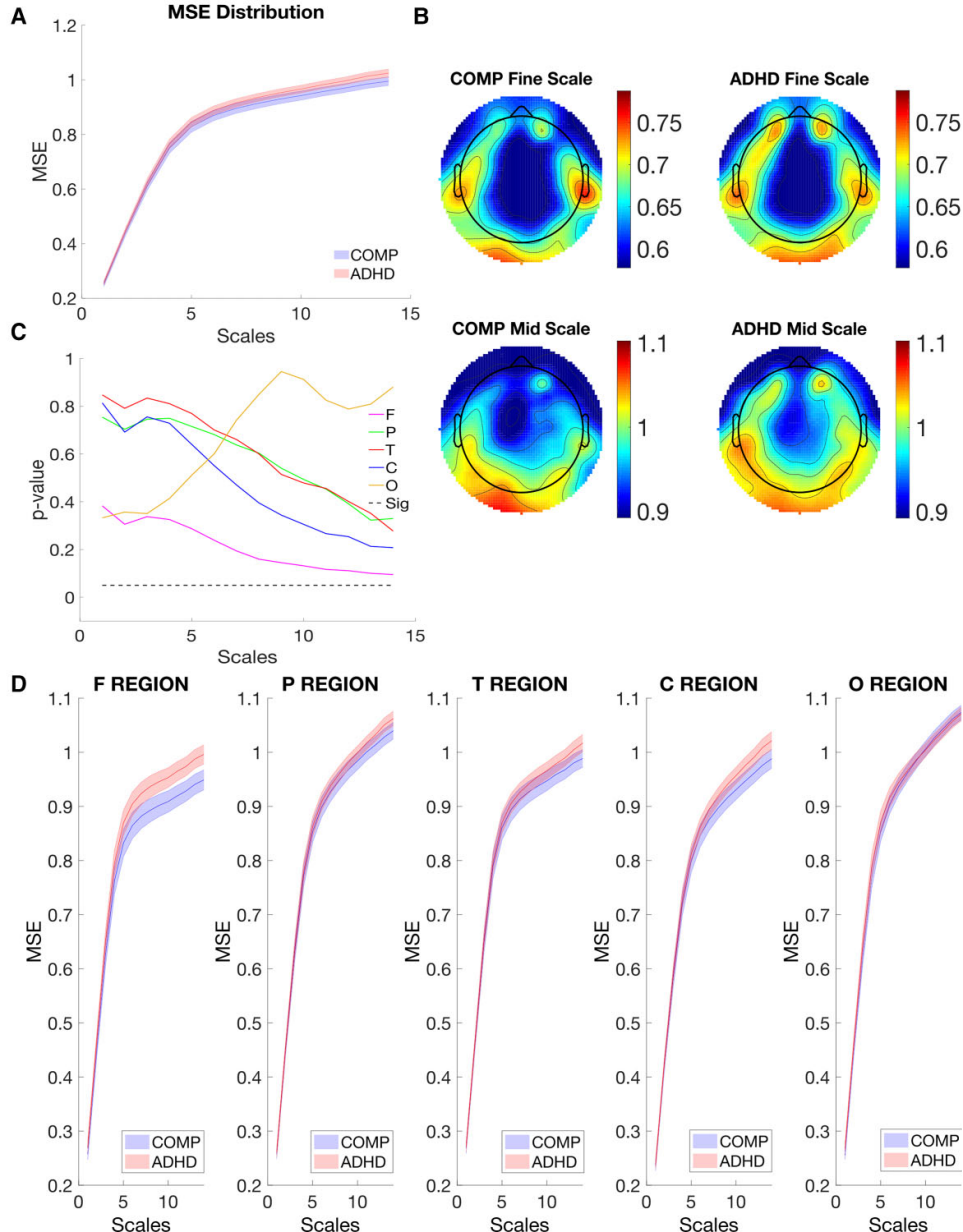
**Temporal and spatial comparison of MSE in the active task state of EEG.** (**A**) The MSE comparison over scales. MSE from the comparison and ADHD group were averaged over all sites and compared over scales (1–14), with standard error as a shaded area. (**B**) The topographic comparison of MSE. MSE was averaged over scales and compared topographically. (**C**) Group difference of MSE between the comparison and ADHD groups. A paired *t*-test of MSE averaged within each brain site was performed over all scales. The Benjamini and Hochberg method (FDR = 0.05) was applied to adjust the *P*-values, which was not significant in the task state. (**D**) MSE comparison over scales within different sites in the active task state. MSE was averaged within each site and compared over the scale with standard error as a shaded area. *Note*: MSE is the multiscale entropy; F Site is the frontal site; P Site is the parietal site; T Site is the temporal site; C Site is the central site; O Site is the occipital site.

First, significant main effects were found for Scale [*F*(1, 58)  =  2273.079, *P* < 0.001, *η*^2^ = 0.975] and Site [*F*(4, 232)  =  6.866, *P* < 0.001, *η*^2^ = 0.225], which suggested MSE was different at different scales or sites as listed in Supplementary Table 2. To illustrate MSE difference, we plotted MSE across different scales, which indicated MSE increased with scale in both the comparison and ADHD groups in Fig. 3A. Further, MSE in frontal and central sites was lower than that in other sites (Supplementary Table 2). The main effect of Group was not significant [*F*(1, 58)  =  0.82, *P* =  0.369, *η*^2^ = 0.014].

Similar to the resting state, the results of the repeated measure ANOVA showed a significant three-way interaction among Group, Site and Scale [*F*(4, 232)  =  7.667, *P* <  0.001, *η*^2^ = 0.117], indicating that differences of MSE between the two groups during the go/nogo task differed at different electrode sites, which also depended on different scales (Fig. 3B and D). The effect suggested MSE from the COMP group was, counter to expectations, lower than that of the ADHD group particularly in the frontal sites at higher scales.

There was no significant two-way interaction found between Scale and Group [*F*(1, 58)  =  0.341, *P* = 0.561, *η*^2^ = 0.006] nor between Site and Group [*F*(4, 232)  =  1.108, *P* =  0.353, *η*^2^ = 0.019], which suggested similar distributions of MSE between the COMP and ADHD groups at any corresponding scale or sites. Interaction between Site and Scale [*F*(8, 464)  =  96.422, *P* < 0.001, *η*^2^ = 0.624] was statistically significant, which suggested, in both groups, MSE has different distribution over scales at different sites.

Despite the significance of the three-way main Omnibus ANOVA, further Benjamini and Hochberg^74^ corrections to adjust *P*-values (FDR = 0.05) indicated that none of the *post hoc* effects survived significance. Our hypothesis was therefore not confirmed. In fact, the pattern of results suggests that during task states, MSE was higher in the ADHD group.

**Figure 4:**
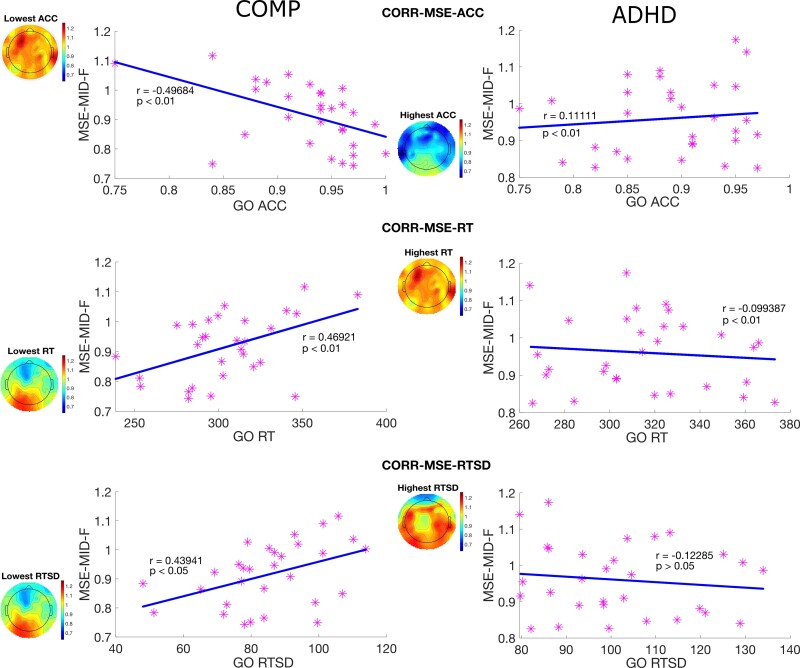
**Correlation between MSE in the active task state and task performance.** Scatter plots with a regression line between MSE averaged over mid scales at the frontal site and three task performance measurements, including ACC for go task, RT for go task and RTSD for go task. The topoplots represent the MSE distribution corresponding to extreme points of task performance measurements in a significant correlation. *Note*: CORR-MSE-ACC is the correlation between MSE and the ACC; CORR-MSE-ACC is the correlation between MSE and the RT; CORR-MSE-RTSD is the correlation between MSE and the RTSD; COMP is the comparison group; ADHD is the ADHD group.

Finally, we also found a significant negative correlation between MSE and task performance in the comparison group as shown in Fig. 4, but not in the ADHD group. Increased MSE across mid scales in the frontal site was associated with lower ACC, larger RT and larger RTSD. Furthermore, correlation coefficients between MSE and task performance (ACC, RT and RTSD) were compared between the comparison and ADHD groups. The Fisher’s *z*-scores were −2.413, 2.237 and 2.186 (*P* = 0.008, 0.013 and 0.014), indicating these relations were significantly different between the comparison group and ADHD group.

### MSE-Δ from the resting state to the active task state

Our third hypothesis stated that the MSE transition (MSE-Δ) from the resting state to the task state would be larger in the comparison group.

**Figure 5:**
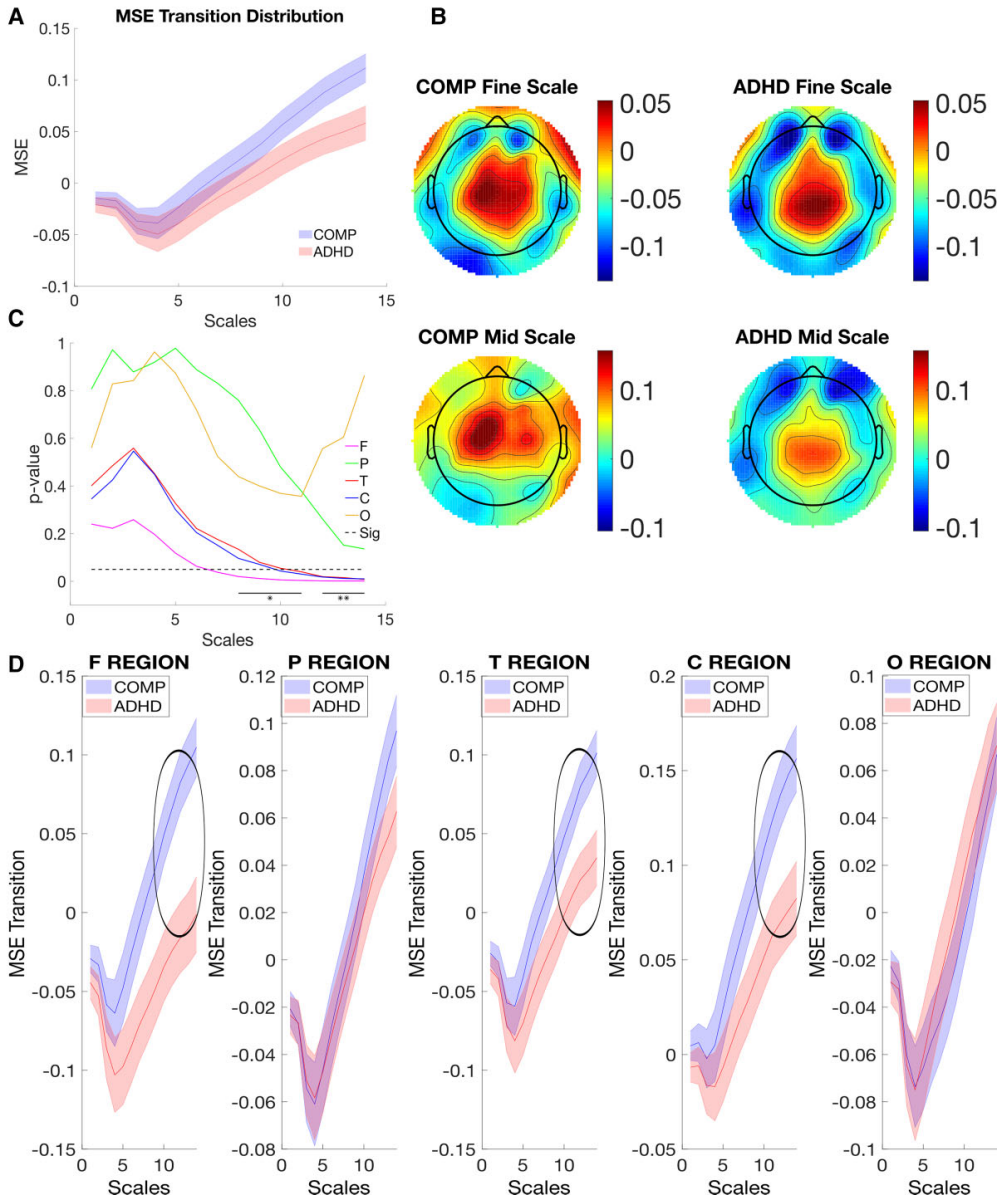
**Temporal and spatial comparison of MSE transition of EEG.** (**A**) Comparison of MSE-Δ over scales. MSE transitions between the comparison and ADHD groups were averaged over all sites and compared over scales (1–14), with standard error as a shaded area. (**B**) The topographic comparison of MSE transition. MSE transitions averaged over scales and compared topographically. (**C**) Group difference of MSE between the comparison and ADHD groups. Paired *t*-test was performed for MSE transition averaged within each site over all scales. The Benjamini and Hochberg method (FDR = 0.05) was applied to adjust the *P*-values, whose significance was marked by line segments with stars (*P < 0.05; **P < 0.01). (**D**) Comparison of MSE transition over scales within different sites. MSE was averaged within each site and compared over scales with standard error as a shaded area. *Note*: MSE is the multiscale entropy; F Site is the frontal site; P Site is the parietal site; T Site is the temporal site; C Site is the central site; O Site is the occipital site.

First, significant main effects were found for Scale [*F*(1, 58) =  179.309, *P* <  0.001, *η*^2^ = 0.756] and Site [*F*(4, 232)  =  18.950, *P* <  0.001, *η*^2^ = 0.246] which suggested different MSE-Δ at different scales or sites as shown in Supplementary Table 3. To illustrate MSE-Δ, we plotted MSE-Δ across different scales, which indicated MSE-Δ decreased within fine scales and increased across mid scales in both comparison and ADHD groups in Fig. 5A. MSE-Δ in central sites are higher than that in other sites (Supplementary Table 3). The main effect of Group was not significant [*F*(1, 58) =  2.022, *P* =  0.160, *η*^2^ = 0.034].

However, similar to the resting and task states, the results of the repeated measure ANOVA also showed a significant three-way interaction of MSE-Δ among Group, Site and Scale [*F*(4, 232)  =  11.595, *P* <  0.01, *η*^2^ = 0.167], indicating that differences of MSE-Δ between the two groups differed at different electrode sites, which also depended on different scales (Fig. 5B and D). To examine MSE-Δ in a more detailed manner, we compare MSE-Δ at different sites over different sites, which indicated MSE-Δ for ADHD was indeed found to be lower (FDR = 0.05) than that for their peers in frontal, temporal and central sites over mid scales as shown in Fig. 5C and D.

There were significant two-way interactions found between Scale and Group [*F*(1, 58)  =  5.006, *P* <  0.029, *η*^2^ = 0.079] and between Site and Group [*F*(4, 232)  =  4.831, *P* <  0.05, *η*^2^ = 0.077]. Specifically, different MSE-Δ between the comparison and ADHD groups were found at the frontal, temporal and central sites, but not at the parietal and occipital site (Fig. 5D). And while MSE-Δ averaged all sites in the comparison group was larger than that in the ADHD group across mid scales, but not across fine scales as shown in Fig. 5A. Interaction between Site and Scale [*F*(4, 232)  =  11.595, *P* <  0.001, *η*^2^ = 0.167] was also statistically significant, which suggested, in both groups, MSE-Δ has different distribution over scales at different sites.

**Figure 6:**
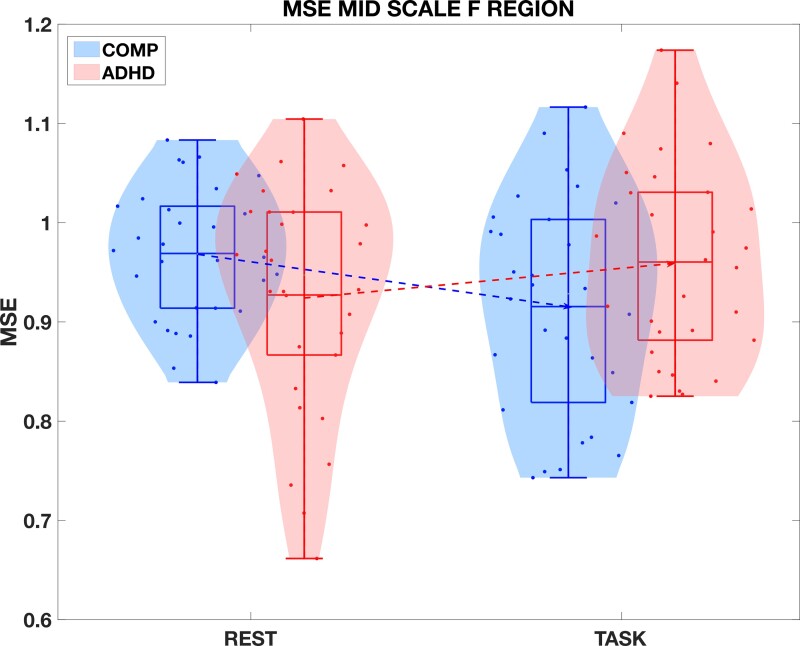
**The overall effect of MSE under different states from the comparison and ADHD groups.** A one-way repeated measures ANOVA was conducted to compare the effect of different states on MSE in the comparison and ADHD groups. There was a significant effect of different states on the group (*P* = 0.004). *Note*: MSE is the multiscale entropy; REST is the resting state; TASK is the active task state; MSE MID SCALE F Site is the MSE averaged within mid scales over the frontal site; COMP is the comparison group; ADHD is the ADHD group.

When visualizing MSE from the frontal site within the mid scales in Fig. 6, there was a decrease of averaged MSE from the resting state to task state in the comparison group, but an increase of averaged MSE in the ADHD group. There was significant two-way interaction found between Group and State [*F*(1, 116)  =  5.866, *P* <  0.05, *η*^2^ = 0.048], indicating group factors would influence MSE in different states, which is consistent with our finding of MSE-Δ, i.e. the change of MSE from the resting state to the active task state was different between the comparison and ADHD groups.

**Figure 7:**
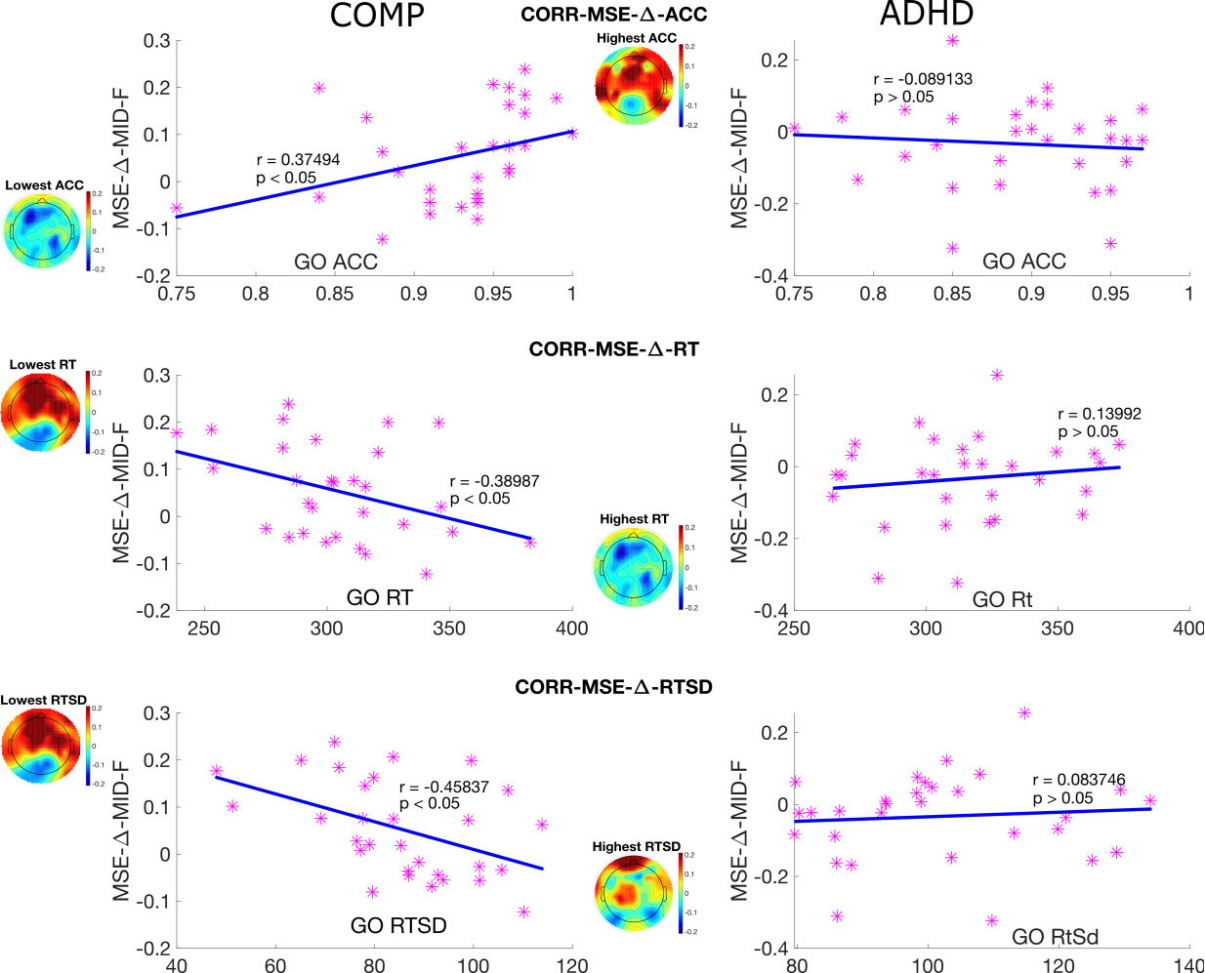
**Correlation between MSE transition and task performance in both groups.** Scatter plots with a regression line between MSE transition averaged over mid scales at the frontal site and three task performance measurements, including ACC for go task, RT for go task and RTSD for go task. *Note*: CORR-MSE-ACC is the correlation between MSE transition and the ACC; CORR-MSE-ACC is the correlation between MSE transition and the RT; CORR-MSE-RTSD is the correlation between MSE transition and the RTSD; COMP is the comparison group; ADHD is the ADHD group.

We also found a significantly negative correlation between MSE-Δ and behaviour measurements in the comparison group as shown in Fig. 7, but not for the ADHD group. Increased MSE-Δ across mid scales in the frontal site was associated with lower ACC (*r* = 0.37494, *P* < 0.05), larger RT (*r* = −0.38987, *P* < 0.05) and a larger RTSD (*r* = −0.45837, *P* < 0.05). Furthermore, correlation coefficients between MSE-Δ and task performance (ACC, RT and RTSD) were compared between the comparison and ADHD groups. The Fisher’s *z*-scores were 1.777, −2.03 and −2.128 (*P* = 050.038,  0.021 and 0.017), indicating these relations were significantly different between the comparison group and ADHD group.

## Discussion

The present study aimed to investigate whether and how adults with ADHD differ from their peers in EEG complexity under different mental states. The ADHD group reported severe impairments in everyday functioning and showed relatively poorer task performance. Our most robust finding was that the transition of MSE, our brain complexity measure, from the resting state to the active task state at the frontal site was larger in the comparison group than in the ADHD group, and correlated with task performance in the comparison, but not the ADHD, group. Findings during the rest and task states were less robust, though the pattern indicated that MSE during the resting state was larger in the comparison group than the ADHD group across coarse scales—an effect that was driven by frontal sites. During the task state, counter to our expectations, the reverse pattern was present. In general, for task states, lower MSE at frontal sites was correlated with better task performance.

Our hypothesis concerning lower MSE in the ADHD group compared with the comparison group during the resting state was confirmed, albeit weakly, for coarse time scales at frontal sites. This could indicate weaker long-range interaction between the frontal site with other sites, respectively, in the ADHD group.^38–40^ The finding was consistent with the executive dysfunction theory, that symptoms of ADHD were associated with abnormalities in frontal–parietal and frontal–striatal circuits.^75^

Previous studies on MSE during the resting state have revealed an increase of MSE along with the development of the brain into adulthood.^40^ Furthermore, smaller MSE during the resting state in the ADHD group could suggest a potential maturational delay of the cortex, which was in line with other behavioural and neuropsychological studies.^76,77^ Smaller MSE across coarse scales in the ADHD group was consistent with earlier findings that showed reduced neural oscillations around the alpha band for adults with ADHD during the resting state.^53^

Smaller MSE within coarse scales at frontal sites in the ADHD group suggests lower itinerancy in distributed neural networks related to frontal sites, which could be associated with lower cognitive flexibility^78^ and difficulty to initialize attention.^20^

Several studies^16,78–81^ suggested that the complexity or neural oscillation of brain activity during the resting state could provide insight into cognitive performance, such as sensitivity to stimuli and cognitive flexibility. Regarding the relationships between behaviour measurements and MSE across different scale ranges at different brain sites, no significant correlation could be found. Although a significant relationship between brain entropy in resting state and task performance has been found in Saxe *et al*.,^82^ different entropy measurements and task requirements could explain the lack of significant correlation in the current study. The implementation of cognitive flexibility would require the coherent act of several executive function subdomains including inhibitory control,^11^ which is evaluated during go/nogo task. If the MSE in the resting state is more related to cognitive flexibility, it might not be sensitive to a go/nogo task.^83^

Our second hypothesis, predicting that the ADHD group would have lower MSE than the comparison group during an active state, was not confirmed. In fact, there was a three-way interaction showing an opposite effect suggesting, albeit not confirmed after correction for multiple comparisons, that the ADHD group has higher MSE during active states for frontal sites at coarse scales than the comparison group.

When examining the overall pattern of results, a higher MSE during the task state for the ADHD group may indicate that increased brain complexity during highly constrained and predictable task conditions (such as a go trail in a go/nogo) may not be beneficial. Under such task conditions, a high convergence and predictability of brain system dynamics, as reflected by low itinerancy, may mediate a more focused performance. In other words, neural systems being in a less flexible, more rigid state, are more efficient to meet more constrained task demands.

This interpretation is consistent with our task performance findings suggesting that lower MSE during task conditions was actually associated with better task performance. That this correlation was only there for the comparison group may suggest that this group was able to leverage their MSE to achieve better performance whereas this was not the case for individuals with ADHD.

Our third hypothesis was confirmed: smaller MSE transitions from the resting state to the active task state (MSE-Δ) within mid scales at frontal sites characterized individuals with ADHD. Smaller MSE-Δ in the ADHD group indicated their brain could not generate sufficient changes of long-range interaction between frontal sites with other regions for cognitive tasks, which was consistent with the executive dysfunction theory for ADHD.^75^ In other words, the neural activity from participants in the ADHD group is likely to be less flexible in adjusting to process tasks.

Previous studies on MSE-Δ revealed that there was an increase of MSE-Δ along with the maturation of the brain.^80^ Furthermore, smaller MSE-Δ in the ADHD group could be indicative of a potential maturational delay of the brain,^76,77^ which was consistent with previous findings of smaller MSE in the ADHD group during the resting state.

This effect, since it was a difference measure, was not solely driven by the resting state. As previously discussed, the MSE from the comparison group was larger than that from the ADHD group (statistically significant) in the resting state but the MSE from the comparison group was smaller than that from the ADHD group in the active task state. MSE in both resting and task states contributed to the MSE difference measurement as shown in Fig. 6. Put differently, individuals with ADHD appeared less able to switch from task states, where low itinerancy was demanded, to resting states, where high itinerancy appeared more beneficial.

MSE-Δ could provide insight into task-related functional connectivity changes and evaluate dynamic itinerancy change within certain neural networks.^84,85^ When the relationships between MSE-Δ and task performance were investigated, MSE-Δ in the comparison group within mid scales at frontal sites were found positively correlated with ACC for go trials, negatively correlated with RT for correct go trials and negatively correlated with the RTSD for correct go trials. Such relationships were not found in the ADHD group, which could be related to abnormal functional connectivity associated with MSE across mid scales at frontal sites during the active task state.

Our study also compared performance measures in the go/nogo task (ACC, RT and RTSD) between the comparison and ADHD groups. Consistent with previous literature, the ACC from individuals with ADHD was lower than that from their healthy peers. The relatively small effect size of group differences in ACC can be explained by the nature of the sample: college students with ADHD. These subjects are relatively high functioning adults with ADHD, especially in their performance on standardized neuropsychological tests.^86^ The lack of group differences in RT could be explained by similar reasons.

Compared with healthy subjects, the RTSD from adults with ADHD was larger than that from their peers with a medium effect size. In other words, at a behavioural level, the ADHD group represented increased intra-individual variability in RT for go/nogo tasks. A previous study^87^ investigated the RTSD in individuals with ADHD during different tasks and linked RTSD to distractibility, which suggested RTSD could be a valid measure of inattention in ADHD.

Several limitations of the current study are noteworthy. First, our sample size was relatively small, which could potentially reduce the power of this study. A correction for multiple comparisons suggested that the interpretation of the ANOVA effect for the rest and task states should be taken with caution, however, a larger sample size could have confirmed this finding and increased confidence in some of our findings pertaining to the rest and task states. This is something we hope future studies can provide more clarity on. Second, we did not employ the random sampling procedure for participant selection but used the convenience sampling method. Because our research was only done on college students, we will caution readers to readily extend our findings to the general population. Third, the experiment sessions were about 3 h, which might make participants tired and induce additional noise in the EEG recording. Although the authors believe the effects of fatigue on the data to be minimal on the basis of the experience of research assistants and procedures put in place combating fatigue, we cannot exclude its effects. Finally, we would like to make the reader aware that, although the eyes-closed condition was considered a cleaner^88^ and more discriminate measure^53^ of MSE than the eyes-open condition in our sample, the eyes-open condition may have had an advantage as it can function as a better control when comparing active and passive conditions.

## Conclusion

To the best of our knowledge, this is one of the few studies of EEG complexity for college students with ADHD. The study provides support for the idea that MSE can evaluate complex brain dynamics in subjects with ADHD, provide insight into neural connectivity and their relationships with ADHD symptoms. Findings suggest that the brains of individuals with ADHD have lower cognitive flexibility in the resting state than their healthy peers, as well as lower cognitive stability in the active task state. The brain’s ability to change from a resting state to an active state is represented by MSE transition and significantly different between individuals with ADHD and their peers. The interpretation of the MSE complexity over different mental states could provide insight into the neural mechanism of ADHD.

## Supplementary Material

fcac054_Supplementary_Data

## Abbreviations

ACC =: accuracy
ADHD =: attention-deficit/hyperactivity disorder
ASRS =: Adult ADHD Self Report Scale
CFQ =: Cognitive Failures Questionnaire
DSM =: Diagnostic and Statistical Manual of Mental Disorders
EGI =: Electrical Geodesic Inc.
FDR =: false discovery rate
ICA =: Independent Component Analysis
MSE =: multiscale entropy
RT =: reaction time
RTSD =: standard deviation of reaction time
