# Supplementary material for: Electroencephalography complexity in resting and task states in adults with attention-deficit/hyperactivity disorder
Source: Brain Commun. 2022 Mar 7;4(2):fcac054. doi: 10.1093/braincomms/fcac054 (PMC8971899; doi:10.1093/braincomms/fcac054)
Supplement: fcac054_Supplementary_Data [file fcac054_supplementary_data.docx]

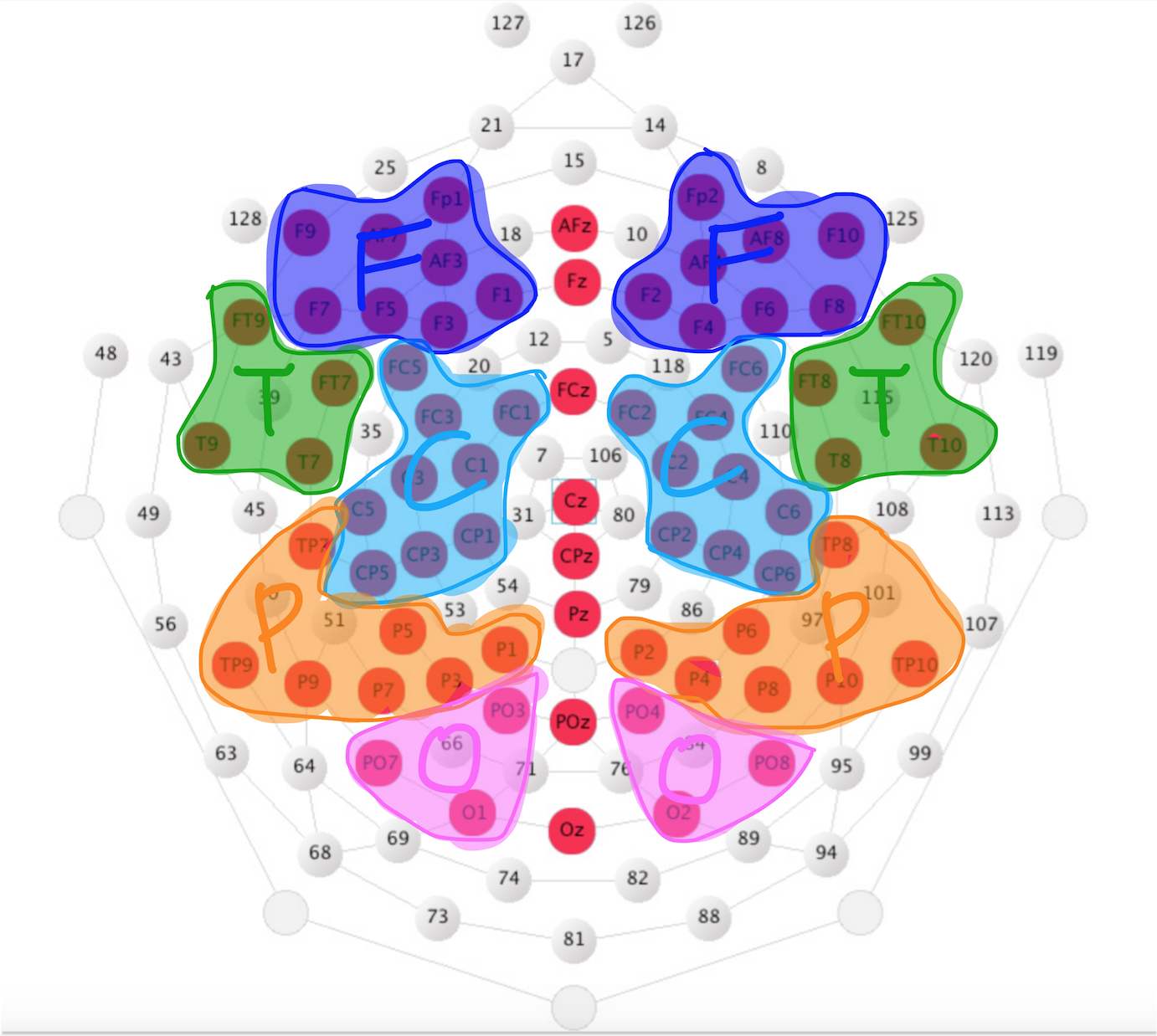


**Supplementary Figure 1** **Electrode clusters represent corresponding brain sites.** Deep blue represents the Frontal Site (F Site). Green represents the Temporal Site (T Site). Light blue represents the Central Site (C Site). Orange represents the Parietal Site (P Site). Pink represents the Occipital Site (O Site)represents

**Supplementary Table 1 Descriptive for MSE in the resting state**

|  |  | Fine  Scale | | Mid Scale | | Coarse  Scale | | Overall  Scale | |
| --- | --- | --- | --- | --- | --- | --- | --- | --- | --- |
|  |  | M | SD | M | SD | M | SD | M | SD |
| Frontal  Site | COMP | 0.59 | 0.059 | 0.97 | 0.066 | **1.09** | 0.075 | 0.89 | 0.057 |
|  | ADHD | 0.58 | 0.072 | 0.93 | 0.113 | **1.03** | 0.133 | 0.85 | 0.100 |
| Parietal  Site | COMP | 0.60 | 0.066 | 1.03 | 0.059 | 1.17 | 0.049 | 0.95 | 0.045 |
|  | ADHD | 0.61 | 0.064 | 1.03 | 0.077 | 1.16 | 0.096 | 0.94 | 0.067 |
| Temporal  Site | COMP | 0.62 | 0.065 | 1.01 | 0.060 | 1.13 | 0.057 | 0.93 | 0.051 |
|  | ADHD | 0.60 | 0.076 | 0.97 | 0.094 | 1.08 | 0.113 | 0.89 | 0.085 |
| Central  Site | COMP | 0.62 | 0.065 | 1.05 | 0.049 | 1.18 | 0.039 | 0.96 | 0.037 |
|  | ADHD | 0.60 | 0.072 | 1.02 | 0.101 | 1.14 | 0.117 | 0.93 | 0.088 |
| Occipital  Site | COMP | 0.59 | 0.068 | 1.02 | 0.065 | 1.18 | 0.046 | 0.94 | 0.047 |
|  | ADHD | 0.61 | 0.063 | 1.03 | 0.085 | 1.18 | 0.094 | 0.95 | 0.070 |
| Overall  Site | COMP | 0.60 | 0.599 | 1.01 | 0.049 | 1.14 | 0.045 |  |  |
|  | ADHD | 0.60 | 0.061 | 0.99 | 0.085 | 1.11 | 0.103 |  |  |

Note: COMP is the comparison group; ADHD is the ADHD group; Fine Scale is from 1 to 6; Mid Scale is from 7 to 14; Coarse Scale is from 15 to 20; M and SD are used to represent the mean and standard deviation, respectively; The locations of Frontal Site, Parietal Site, Temporal Site, Central Site, and Occipital Site were shown in Figure 1; Bolded text in the table indicate there’s a significant difference between the COMP and ADHD at corresponding sites and scales.

**Supplementary Table 2 Descriptive for MSE in the task state**

|  |  | Fine Scale | | Mid Scale | | Overall Scale | |
| --- | --- | --- | --- | --- | --- | --- | --- |
|  |  | M | SD | M | SD | M | SD |
| Frontal Site | COMP | 0.63 | 0.106 | 0.92 | 0.108 | 0.79 | 0.104 |
|  | ADHD | 0.66 | 0.100 | 0.96 | 0.098 | 0.83 | 0.095 |
| Parietal Site | COMP | 0.64 | 0.084 | 0.99 | 0.085 | 0.84 | 0.081 |
|  | ADHD | 0.65 | 0.082 | 1.01 | 0.066 | 0.85 | 0.066 |
| Temporal Site | COMP | 0.65 | 0.098 | 0.95 | 0.096 | 0.82 | 0.094 |
|  | ADHD | 0.66 | 0.085 | 0.97 | 0.084 | 0.84 | 0.079 |
| Central Site | COMP | 0.60 | 0.090 | 0.94 | 0.098 | 0.79 | 0.092 |
|  | ADHD | 0.61 | 0.073 | 0.96 | 0.082 | 0.81 | 0.070 |
| Occipital Site | COMP | 0.64 | 0.077 | 1.01 | 0.074 | 0.85 | 0.071 |
|  | ADHD | 0.66 | 0.089 | 1.01 | 0.066 | 0.86 | 0.071 |
| Overall Site | COMP | 0.62 | 0.081 | 0.94 | 0.087 |  |  |
|  | ADHD | 0.63 | 0.072 | 0.96 | 0.071 |  |  |

*Note*: COMP is the comparison group; ADHD is the ADHD group; Fine Scale is from 1 to 6; Mid Scale is from 7 to 14; M and SD are used to represent the mean and standard deviation, respectively; The locations of Frontal Site, Parietal Site, Temporal Site, Central Site, and Occipital Site were shown in Figure 1.

**Supplementary Table 3 Descriptive for MSE transition in the task state**

|  |  | Fine Scale | | Mid Scale | | Overall Scale | |
| --- | --- | --- | --- | --- | --- | --- | --- |
|  |  | M | SD | M | SD | M | SD |
| Frontal Site | COMP | -0.04 | 0.092 | **0.05** | 0.102 | 0.01 | 0.095 |
|  | ADHD | -0.08 | 0.103 | **-0.03** | 0.121 | -0.05 | 0.109 |
| Parietal Site | COMP | -0.04 | 0.075 | 0.04 | 0.082 | 0.01 | 0.076 |
|  | ADHD | -0.04 | 0.076 | 0.02 | 0.075 | 0.00 | 0.071 |
| Temporal Site | COMP | -0.04 | 0.079 | **0.05** | 0.083 | 0.01 | 0.078 |
|  | ADHD | -0.06 | 0.088 | **0.00** | 0.092 | -0.02 | 0.087 |
| Central Site | COMP | 0.01 | 0.083 | **0.11** | 0.096 | 0.07 | 0.088 |
|  | ADHD | -0.01 | 0.079 | **0.05** | 0.097 | 0.03 | 0.085 |
| Occipital Site | COMP | -0.05 | 0.073 | 0.01 | 0.088 | -0.02 | 0.078 |
|  | ADHD | -0.05 | 0.092 | 0.02 | 0.107 | -0.01 | 0.098 |
| Overall Site | COMP | -0.02 | 0.066 | 0.06 | 0.077 |  |  |
|  | ADHD | -0.03 | 0.073 | 0.03 | 0.082 |  |  |

*Note*: COMP is the comparison group; ADHD is the ADHD group; Fine Scale is from 1 to 6; Mid Scale is from 7 to 14; M and SD are used to represent the mean and standard deviation, respectively; The locations of Frontal Site, Parietal Site, Temporal Site, Central Site, and Occipital Site were shown in Figure 1.
